# Can Dendritic Cell Vaccination Prevent Leukemia Relapse?

**DOI:** 10.3390/cancers11060875

**Published:** 2019-06-22

**Authors:** Liam J. O’Brien, Camille Guillerey, Kristen J. Radford

**Affiliations:** Mater Research Institute, The University of Queensland, Translational Research Institute, Woolloongabba, QLD 4102, Australia; liam.obrien@mater.uq.edu.au (L.J.O.); c.guillerey@uq.edu.au (C.G.)

**Keywords:** leukemia, dendritic cell, vaccination, CD141, moDC, CD1c, immunotherapy

## Abstract

Leukemias are clonal proliferative disorders arising from immature leukocytes in the bone marrow. While the advent of targeted therapies has improved survival in certain subtypes, relapse after initial therapy is a major problem. Dendritic cell (DC) vaccination has the potential to induce tumor-specific T cells providing long-lasting, anti-tumor immunity. This approach has demonstrated safety but limited clinical success until recently, as DC vaccination faces several barriers in both solid and hematological malignancies. Importantly, vaccine-mediated stimulation of protective immune responses is hindered by the aberrant production of immunosuppressive factors by cancer cells which impede both DC and T cell function. Leukemias present the additional challenge of severely disrupted hematopoiesis owing to both cytogenic defects in hematopoietic progenitors and an abnormal hematopoietic stem cell niche in the bone marrow; these factors accentuate systemic immunosuppression and DC malfunction. Despite these obstacles, several recent clinical trials have caused great excitement by extending survival in Acute Myeloid Leukemia (AML) patients through DC vaccination. Here, we review the phenotype and functional capacity of DCs in leukemia and approaches to harness DCs in leukemia patients. We describe the recent clinical successes in AML and detail the multiple new strategies that might enhance prognosis in AML and other leukemias.

## 1. Introduction

Dendritic Cells (DCs) are professional antigen-presenting cells (APCs) whose primary role is to process and present antigens to B and T lymphocytes to induce adaptive immunity [1]. DCs mature upon encounter with various environmental cues, such as microbe fragments or necrotic cell products, present antigen highly efficiently and secrete a range of cytokines and chemokines to mediate sustained immune activation at sites of infection or within tumors. In addition to DCs’ primary role in priming anti-tumor T cells, there is increasing evidence that cross-talk between Natural Killer (NK) cells and DCs is instrumental to the development of anti-tumor responses [2,3,4].

**Figure 1:**
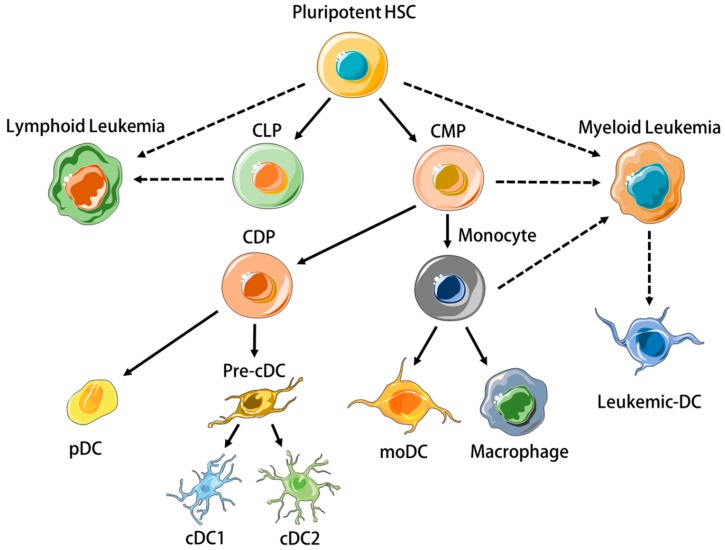
Plasmacytoid, conventional, and monocyte-derived dendritic cells (DCs) differentiate from distinct progenitors. Oncogenic mutations in hematopoietic progenitor cells may result in their clonal proliferation and the pathogenesis of leukemia. Leukemic myeloid cells may differentiate into cells with DC properties (Leukemic-DCs). HSC = Hematopoietic Stem Cell, CLP = Common Lymphoid Progenitor, CMP = Common Myeloid Progenitor, CDP = Common Dendritic Cell Progenitor, pDC = plasmacytoid DC, cDC = Conventional DC, moDC = monocyte-derived DC. Cellular art modified from Servier medical art repository under Creative Commons Attribution 3.0 Unported License https://creativecommons.org/licenses/by/3.0/legalcode.

DCs are heterogeneous [5]. Human DC subtypes include conventional DCs (cDCs), plasmacytoid DCs (pDCs), and monocyte-derived DCs (moDC) [1], which all arise from separate hematopoietic precursors (Figure 1) and differ significantly in terms of transcriptome, phenotype and function. This review will focus on CD11c^+^ DCs, i.e., cDCs, and mo-DCs, as these subtypes have been the most utilized in leukemia vaccinations. cDCs can be further divided into CD141^+^ (BDCA3^+^) type 1 cDCs (cDC1) and CD1c^+^ (BDCA1^+^) type 2 cDCs (cDC2). cDC1s have received particular attention because they excel in presenting exogenously-derived cellular antigen to CD8^+^ T cells, a process called cross-presentation that is essential for cancer immunosurveillance [6,7,8]. MoDC differentiate from monocytes under inflammatory situations in peripheral tissues, express several macrophage-associated markers such as CD206, CD14, and CD11b, and secrete IL-6, TNFα, IL-12, and IL-1β ex vivo without restimulation if isolated from tumour ascites [9,10]. MoDC also express CCR7 [11], efficiently activate CD4^+^ and CD8^+^ T cells in vitro [9], and since they can be readily generated from mononuclear cells in vitro using various cytokine cocktails, they are valuable research tools [12].

Leukemias are neoplastic disorders characterised by the clonal proliferation of immature immune cells in the bone marrow (BM) [13]. They are classified as ‘myeloid’ or ‘lymphoid’, depending on the cell precursor from which they originate (Figure 1) [14,15]. As with solid tumors, disease progression occurs despite endogenous immune responses to leukemic cells [16]. The creation of an immunosuppressive micro-environment in the BM is an important feature of leukemias that prevents normal differentiation of nonleukemic hematopoietic stem cells (HSCs) and anti-leukemic immune responses [17,18,19]. Systemic immunosuppression becomes prominent with progressive disease in both lymphoid [20] and myeloid [21] leukemias, involving inhibitory T cell pathways [22], regulatory immune cells [23], and secretion of cytokines and metabolic enzymes such as IL-10 [24], TGFβ [25], and indoleamine-2,3-dioxygenase (IDO) [26].

The paradigm for solid tumors is that T cell priming occurs primarily in the tumor-draining lymph node, although it might also take place within the tumor bed [27]. In leukemia, it is not known whether T cell priming occurs in the BM or in disseminated locations around the body. It should be noted that, even if recirculating mature DCs homing to the BM elicit potent central memory responses [28], the abundance of immunosuppressive blasts and/or myeloid-derived suppressor cells (MDSCs) in the BM might preclude T cell priming. In both solid tumours and leukemias, the induction of MDSCs is a hallmark feature of immunosuppression [29,30].

In this review, we will detail how leukemia affects DC homeostasis and function and thereby prevents the development of effective T cell responses. Moreover, we will discuss different therapeutic strategies targeting DCs in hematological cancers, their ability to restore effective anti-tumor immunity and their clinical efficacy.

## 2. Dysregulation of DC Homeostasis and Function in Leukemia

### 2.1. DCs in Myeloid Leukemias

Myeloid leukemias are classified into Acute Myeloid Leukemia (AML) and Chronic Myeloid Leukemia (CML). AML is defined by an infiltration of the BM by immature blast cells superior to 20% while CML is characterised the bcr/abl fusion protein resulting from the translocation t(9:22) [31]. The co-existence of cytogenetically ‘normal’ cDC and moDC as well as leukemia-derived DCs (leukemic-DCs) expressing the bcr/abl fusion protein has been described in vivo in CML patients, and proportions vary among patients and DC subsets: Orsini et al. reported 50–70% of leukemic-DCs among circulating CD1c^+^ cDC2 [32], while Dong et al. showed that 70–95% of moDCs were leukemia-derived [33]. In AML, the identification of leukemic-DC is only feasible in patients with detectable cytogenetic abnormalities, and the separation of cytogenetically normal and leukemic-DC is made difficult by the greatly reduced frequency of DC at diagnosis [34]. However, in AML patients all lineage-negative cells expressing the myeloid and cDC marker CD11c display leukemia-specific cytogenetic abnormalities, indicating their derivation from the malignant clone [35]. Leukemic-DCs from AML and CML patients resemble myeloblasts upon microscopic evaluation [34] and express low levels of HLA-DR [36].

A recent study using an injected mouse AML cell line established that non-leukemic cDC1s were solely responsible for acquiring and cross-presenting leukemia-associated-antigen (LAAs) to CD8^+^ T cells [37]. Cross-presentation of LAAs by cDC1s in this model largely resulted in T cell tolerance until the TLR3 adjuvant polyinosinic-polycytidylic acid (PIC) was administered to mice, supporting the idea that DC function may be rescued in myeloid leukemia.

#### 2.1.1. DCs in AML

Several groups have reported highly variable cDC numbers in the blood or BM of AML patients [35,36,38,39]. While Rickmann et al. linked high CD11c^+^ cDC counts with the presence of the FLT3 internal tandem duplication (FLT3-ITD) present in 30% of AML patients [36], other groups observed no correlation of DC counts with this mutation [38,39], or made no comment [35]. It is likely that FLT3-ITD contributes to increased proliferation of DC precursors as was shown in a mouse model by Lau et al. [40], but maturation of cDC subsets is altered in active AML due to cytogenetic aberrations or an abnormal cytokine milieu, which may induce an MDSC-like phenotype in DC precursors and prevent their final maturation. The highly variable cDC quantity observed in AML patients is explained by the aberrant expression of DC-associated markers on leukemic blasts, as well as overlapping surface markers with MDSCs and monocytes [41]. This was resolved recently by Hsu et al. who utilized a stringent flow cytometry gating strategy with extra lineage and cDC-specific markers to eliminate leukemic blasts and concluded to a dramatic decrease in the blood CD141^+^ cDC1 and CD1c^+^ cDC2 subsets at AML diagnosis [34]. This demonstrated the inclusion of immature DC precursors or blasts expressing DC antigens in the DC gate of the other studies.

There is very limited information about DC functionality in AML at diagnosis. Most groups have focused on optimizing protocols to obtain leukemic-DCs from AML blasts [42,43,44,45,46]. Most studies used IL-4 and GM-CSF, sometimes supplemented with TNFα or other factors. For instance, leukemic-DC differentiation can be enhanced by adding the histone deacetylase inhibitor bryostatin to the cytokine cocktail [47] or by inhibiting Signal Transducer and Activator of Transcription 3 (STAT3) [48]. AML-derived leukemic-DCs stimulate allogeneic T cells in mixed lymphocyte reaction (MLR) assays and generate autologous anti-leukemic CTL responses in vitro [43,45,49,50]. However, PD-L1 and IDO-1 expression by leukemic-DCs might favor T cell tolerance [51,52]. The direct comparison of in vitro derived moDCs from AML-remission patients with matched leukemic-DCs derived from AML blasts at diagnosis revealed that both DCs are able to induce strong allogeneic T cell proliferation [53]. Nevertheless, for 2/3 patients, leukemic-DCs were found less potent than remission moDCs.

#### 2.1.2. DCs in CML

Both cDC1 and cDC2 subsets are reduced in the blood of CML patients [54]; and low DC numbers have been associated with high plasmatic levels of VEGF [54] and with low numbers of circulating CD34^+^CD38^-^ HSCs [55]. A significant body of work indicates that in vitro derived moDCs are dysfunctional in CML [32,33,56]. The altered organization of the actin cytoskeleton of CML in vitro moDCs might explain their rounded morphology and their altered migratory response to MIP-1α [33]. Although unstimulated moDC from CML patients and healthy donors express similar levels of co-stimulatory molecules [33,56], decreased CD80, CD83 and CD40 expression have been observed on cDC2s isolated from CML patients [32]. Moreover, CML moDC maturation after Lipopolysaccharide (LPS) treatment in vitro is defective and is associated with reduced production of IL-8 and TNFα [56].

cDC2s [32] and in vitro derived moDCs from CML patients efficiently induce allogeneic T cell proliferation once activated [33,56,57], but contradictory data have been reported regarding their ability to induce antigen-specific responses. An early study indicated that CML-derived DCs could induce the proliferation of autologous T cells when pulsed with whole tetanus toxin antigen but this study did not provide a comparison with healthy donor DCs nor assessed the specificity of the proliferating cells [57]. By contrast, another study reported that in vitro derived moDCs from CML patients pulsed with whole tetanus toxin antigen failed to induce the proliferation of an antigen specific CD4^+^ T cell clone [33]. Importantly, in this last study, peptide-pulsed CML moDCs did stimulate clonal T responses [33]. These data point to defects in the antigen presentation machinery of CML moDCs and may partly be explained by the decreased ability of CML moDCs to uptake antigens in vitro [33,56].

### 2.2. DCs in Lymphoid Leukemias

The majority of lymphoid neoplasms can be divided into acute (Acute Lymphoblastic Leukemia; ALL) and chronic (Chronic Lymphocytic Leukemia; CLL) subsets. ALL is defined by > 20% of lymphoblasts in the blood, and pathogenesis involves primarily B cell precursors (B-ALL, 75% of cases) or T cell precursors (T-ALL, 25% of cases) [58]. CLL is characterised by the expansion of CD5^+^CD23^+^ B cells in the BM, blood, and secondary lymphoid tissues [59,60].

#### 2.2.1. DCs in ALL

Patients with B-ALL display a severe reduction of cDCs in both the blood and the BM compared to healthy donors [61,62,63]. This defect might be caused by a blockage in hematopoiesis, as suggested by the incapacity of CD34^+^ HSCs isolated from the blood of B-ALL patients to differentiate into cDCs and moDCs under cytokine stimulation in vitro [62], and the aberrant expression of myeloid markers on the leukemic blasts of patients lacking DCs [63]. Compared with B-ALL, cDCs are much less affected in T-ALL and have been reported as slightly reduced [61], increased (in a cohort of pediatric patients) [63], or comparable to healthy donors [62]. Studies on larger cohorts should help reconcile these conflictive results and determine whether differences exist between adult and pediatric T-ALL.

Preliminary data obtained with moDCs generated from an infant diagnosed with B-ALL indicated that in vitro, B-ALL-associated DCs can induce autologous responses when loaded with tumor lysate [64]. Along the same line, moDCs generated from the peripheral blood of B-ALL patients in complete remission and pulsed with leukemic cell lysate increased the cytotoxicity of T lymphocytes against autologous leukemia cells in vitro for 6/8 patients [65]. Intriguingly, in this study, the two patients with no cytotoxicity experienced disease progression, suggesting that DC ability to induce cytotoxic T cell responses might correlate with clinical outcome. In T-ALL, ex vivo analysis indicated that blood DC maturation levels and the frequencies of cDC1 and cDC2 subsets are similar to those of healthy donors [63]. Functional data is limited to the analysis of in vitro derived moDCs from two T-ALL patients that were able to secrete IL-12p70 in vitro in response to CD40L [62].

#### 2.2.2. DCs in CLL

Circulating CD11c^+^ ILT3^+^ cDCs are significantly reduced in CLL patients at diagnosis [66]. Only one study has investigated DCs freshly isolated from the blood of CLL patients and reported the inability of a mixed cDC/pDC population to induce proliferative T cell responses in allo-stimulatory MLR assays [67]. So far, all other studies have focused on in vitro derived moDCs. Early studies reported that in vitro derived moDCs from CLL patients express normal levels of costimulatory molecules and stimulate allogeneic and autologous T cell responses [68,69,70,71]. In stark contrast, a recent study analyzing moDCs following LPS activation in vitro reported that the allogeneic responses induced by LPS-activated moDCs from CLL patients were defective and characterised by reduced T cell proliferation and production of IFN-γ and TNF, a process attributed to the deregulation of the negative regulator SOCS5 [72]. Finally, the majority of the reports indicate a skewed cytokine profile of in vitro derived CLL moDCs, although data are conflicting regarding the exact nature of these defects [68,69,70,72]. The direct inhibition of DC development and functionality by leukemic cells has also been evidenced in CLL where Orsini et al. found that the addition of autologous tumor cells to in vitro cultures decreased DC differentiation [73]. The same group showed that healthy moDC in transwell cultures with CLL cells displayed reduced IL-12 production and this was reversible through neutralization of IL-6, IL-10, and VEGF [67].

### 2.3. Concluding Remarks on DCs in Leukemia

**Figure 2:**
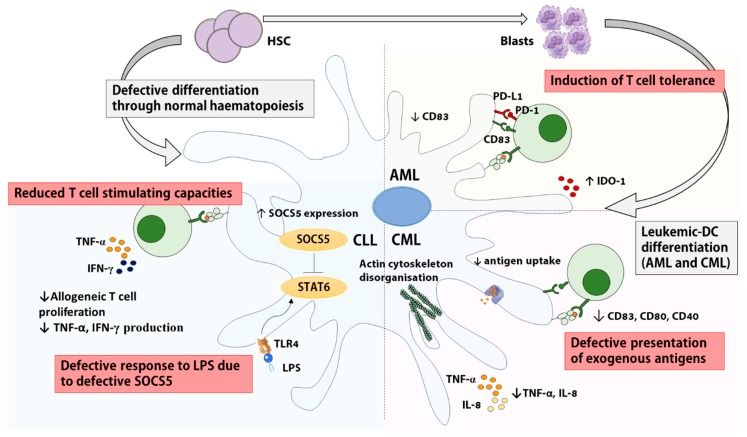
Impairments to conventional and monocyte-derived dendritic cell function in leukemia. DCs exhibit reduced capacity to stimulate T cells in different subsets of leukemia via reduced co-stimulatory molecule expression (Chronic Myeloid Leukemia (CML), Acute Myeloid Leukemia (AML)) [48,51], increased expression of anti-inflammatory cytokines (CLL, AML) [52,72], aberrant actin organization and reduced antigen uptake (CML) [33], disruption of LPS in vitro response through increased SOCS5 expression (CLL) [72], as well as defective differentiation from HSCs (ALL, CLL) or leukemic blasts (AML, CML). IDO1: indoeamine 2,3 dioxygenase. LPS: Lipopolysaccharide. PD-1: Programmed-Death-1. TLR: Toll-like Receptor.

Overall, significant alterations of cDC and moDC numbers and/or functions were observed in most leukemias, except for T-ALL (summarized in Figure 2). The reasons for these defects are multiple and may include a BM failure associated with a depleted pool of nonleukemic HSC [19,55], the inability of HSC to differentiate into DCs [62,63] and the secretion of immunosuppressive cytokines by leukemic cells [73,74]. Interestingly, cDC and moDC deregulation seems to revert, at least partially, once the tumor has been cleared via chemotherapy and/or BM transplant. Indeed, the defective allogeneic stimulatory capacity of moDCs from CLL patients with active disease is no longer observed in CLL patients in remission [73]. Similarly, DC numbers recover to levels within control range in remission AML [34,39] and B-ALL patients [61].

It should be noted that many of the studies reviewed here have been performed in the early 2000’s when the poor knowledge of DC subsets, the paucity of DC-specific reagents and the limitations of flow cytometric systems might have led to incorrect conclusions. Studies on large cohorts using cDC-specific functional markers such as CLEC9A, CLEC10A, XCR1, and CD1c along with stringent gating strategies are needed to provide clarity on circulating cDC phenotype and function in the different types of leukemias, as the overwhelming majority of published studies focused on leukemic or monocyte-derived DC in vitro. So far, only one group has investigated the ex-vivo function of circulating DCs, focusing on the cDC2 subset in CML and CLL [32,73]. As a result, very little insight can be gained into the hematopoietic processes leading to cDC absence and malfunction without further studies investigating circulating cDCs freshly isolated from leukemia patients. Owing to the lack of cDCs at diagnosis in most leukemias, this may only be possible in CML and T-ALL patients, but the mechanisms leading to defective DC differentiation from precursor cells could be investigated in B-ALL, CLL, and AML.

Another limitation of many current studies is the absence of distinction between DCs exhibiting chromosomal aberrations derived from the leukemic clone and cytogenetically ‘normal’ non-leukemic cDC and moDC in myeloid leukemias, even though mouse and human data indicate that the abl-bcr mutation might affect leukemic-DC ability to induce T cell responses [33,75]. The field would also benefit from some extensive functional studies analyzing DC responses to different stimuli. Indeed, discrepancy in the literature regarding the functional capacity of patient-derived DCs might be explained by unresponsiveness to a specific stimulation such as the lack of response to LPS of CLL and CML moDCs [56,72] and the AML cell line MUTZ-3 [76].

## 3. DC-Based Immunotherapy in Leukemia

**Figure 3:**
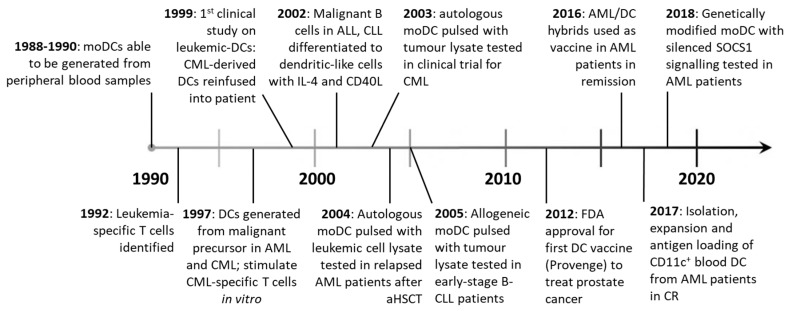
Advances in DC vaccine therapies from 1990–present. After moDCs were able to be generated in vitro in the late 1980s [80] and leukemia specific T cells were identified [81], a host of vaccination strategies have been employed using monocyte or leukemic-derived DCs. So far, only one vaccine (Provenge), containing moDC has been FDA approved for widespread use in patients.

DC vaccination therapies were first developed in the mid 1990’s, after density-gradient centrifugation and in vitro expansion with GM-CSF and IL-4 facilitated the isolation and culture of moDCs from peripheral blood [77,78]. Objective clinical responses were seen in metastatic melanoma patients vaccinated with moDC loaded with tumor antigen ex vivo [79], kick-starting the field of DC vaccination for the treatment of cancer (Figure 3). Currently, two broad strategies exist: patients can receive ex vivo manipulated DCs or be administered DC-targeting drugs aiming to promote DC functions in vivo. Unlike ex vivo DC treatments, DC-targeting drugs are yet to be tested in the clinic.

### 3.1. Ex Vivo Manipulation of DCs for Vaccination

#### 3.1.1. Leukemic-DCs

After the discovery that leukemic-DCs could be differentiated in vitro from AML and CML patient peripheral blood or BM cells [49,82], numerous groups attempted to clinically exploit this phenomenon [83,84,85]. Owing to their derivation from malignant progenitors, leukemic-DCs present a range of known and unknown LAA, thereby making antigen identification and antigen loading unnecessary. Due to the advent of the targeted therapy imatinib in CML, trial recruitments have been cut short and very few CML patients have received leukemic-DCs. Still, data indicate that leukemic-DC vaccination triggered T cell responses in most CML patients, and, in some of them, cytogenetic or clinical responses were observed [86,87,88,89]. Immunological responses were also observed in the few AML patients vaccinated with leukemic-DCs [90,91]; but despite a transient drop in blast numbers in some patients [90], there was no evidence of clinical benefit [91]. In AML, this approach is complicated by the difficulty to generate DCs from every patient [42]. To circumvent this, AML cell lines such as MUTZ-3 [92] and DCOne [93] have been used to generate allogeneic leukemic-DCs. Notably, DCP-001, a novel allogeneic DC vaccine generated from the DCOne AML cell line has shown remarkable results in a cohort of elderly advanced-stage AML patients, with 6/12 patients unexpectedly surviving over 6 months [93]. Patients had to be HLA-matched to at least one allele expressed by DCP-001 (HLA-A2,3, -B44, -DRB1:10,11 and -DQB1:05‚03), and HLA matches between patients and DCP-001 varied from 1 to 5, but interestingly the number of mismatches showed no clear relationship with survival. However, long-term survival was associated with positive T cell responses. For some patients, specific responses against LAA could be detected. Based on these promising data, a multi-center randomized phase II trial in AML patients achieving first complete remission has been set up (NCT03697707).

#### 3.1.2. moDCs

Given that the functional defects of leukemic DCs might diminish their therapeutic efficacy, non-leukemic moDCs might be preferred [53,94]. In myeloid leukemia patients, autologous moDCs are obtained in remission [94]. Autologous moDCs or allogeneic moDC derived from healthy donors have also been investigated for the treatment of lymphoid leukemias [95,96]. An important distinction between monocyte and leukemia-derived DCs is that moDCs do not express leukemic antigens, which necessitates ex vivo antigen loading. The different types of antigens and DC preparation including antigen-loading and maturation/activation methods have been reviewed elsewhere [97,98]. A few studies have compared different antigen-loading methods to generate anti-leukemic T cell responses in vitro. In this regard, loading moDC with both AML-lysate and mRNA was found superior to each method alone, possibly because the dual targeting of class I and class II presentation pathways allows the concomitant priming of CD4^+^ helper T cells that further promote CD8^+^ T cell responses [99]. The fusion of DCs with tumor cells has emerged as a promising approach as it combines the antigen presentation capacity of moDC with the antigen repertoire of leukemic cells [100]. Two groups compared the in vitro CTL priming ability of tumor-DC hybrids with DCs loaded with apoptotic bodies but obtained opposite results: in AML, tumor-DC hybrids where found superior [101] whereas in B-CLL, better results were obtained with apoptotic body-loaded DCs [102]. This may be accounted for by innate differences between AML and CLL fusion hybrids or differing immunosuppressive capabilities of contaminating non-hybridized leukemic cells. Finally, separately loading DCs with different antigens might allow the presentation of multiple antigens while avoiding cellular and molecular antigen competition [103]. MoDCs prepared with this new protocol are now being tested in a phase I/II clinical trial enrolling AML patients in remission (NCT01734304).

To date, in vitro derived moDCs have mostly been used to vaccinate AML [100,104,105] and CLL patients [96,106,107], with only a few ALL patients included in clinical trials [105,108] and no CML patients. This disproportionate distribution is likely due to the availability of imatinib in CML and antibodies targeting malignant B cell antigens in ALL, which have improved prognosis in both diseases [109,110]. Early studies performed with moDCs loaded with a pool of autologous AML-derived peptides [107], tumor lysates [96] or apoptotic bodies [106,111] elicited immunological responses but only modest or no clinical benefit. By contrast, very promising results have been reported recently from several clinical trials, possibly owing to enhanced in vitro activation of DCs, improved culture techniques, and the use of completely novel approaches which facilitate better antigen presentation by DCs in vivo [100]. Further, the inclusion of patients who are not at the end stage of disease may have facilitated better clinical responses [112]. Autologous moDCs electroporated with mRNA encoding the leukemic antigen WT-1 have been administered to a total of 30 AML patients who were at high risk of relapse after induction chemotherapy (NCT00834002 and NCT00965224). Remarkably, this vaccine prevented/delayed relapse in 43% of patients; and clinical responses correlated with increased frequencies of WT-1-specific CD8^+^ T cells [104,113]. Encouraging clinical results have also been obtained by modifying moDCs with an adenoviral vector encoding two LAAs (survivin and MUC1) along with a shRNA moiety to suppress the negative regulator SOCS1 and secretory bacterial flagellin as an adjuvant (NCT01956630). Administration of this genetically modified moDC vaccine to AML and ALL patients compared favorably with donor lymphocyte infusions and induced complete remission in 10/12 AML patients who had relapsed after allogeneic stem cell transplant (SCT) [105]. Unfortunately, immune responses were not measured in this study, and thus could not be correlated with clinical response. The most exciting results of all have come in a recent trial, where vaccination with AML-DC hybrids maintained remission in an average of 71% of AML patients at a median follow-up of 57 months (NCT01096602) [100], more than double the expected survival rate for this disease. An additional clinical trial is underway assessing this strategy in combination with decitabine chemotherapy in post-transplant AML patients (NCT03679650).

#### 3.1.3. cDCs

In spite of their superior cross-presentation and T cell stimulatory capacity compared to moDC [7,114,115], naturally occurring cDCs, and cDC1s in particular, have not been exploited in the vaccination of leukemia patients due to their rarity (cDC1s represent 0.03% of blood mononuclear cells) and lack of suitable isolation methods [116]. However, cDC2s have been isolated via magnetic bead-conjugated CD1c-specific antibodies, activated ex vivo and used to vaccinate melanoma and prostate cancer patients [117,118]. This trial resulted in the generation of tumour-specific CTLs in 4/14 participants. A similar magnetic bead platform can be used to isolate CD141^+^ cDC1s, however this is yet to be tested in the clinic. The ubiquity of CD141 expression in cell types other than cDC1 may pose a problem if using this strategy in leukemia patients.

In addition to cDC-specific isolation techniques, the newly developed anti-CMRF-56 Ab allows the direct isolation of blood APCs including B cells, monocytes and cDCs [119,120]. This technique is feasible to isolate APCs with strong T cell-stimulating capacities from AML patients in remission [34]. Moreover, cutting-edge methods have now been developed to generate large numbers of cDCs in vitro from CD34^+^ progenitor cells and could be used to expand patient cDCs or to generate a bank of allogeneic cDCs for “off the shelf” ex vivo treatment [121,122,123]. Of note, strategies exploiting donor cDCs in the setting of allogeneic SCT merit consideration, since relapse-free survival after allogeneic SCT was correlated with higher counts of donor cDC and pDC per µL of peripheral blood in a cohort comprising pediatric AML and myelodysplastic syndrome patients. This result suggests that expanding donor cDCs and/or pDCs for administration following SCT will enhance response rates [124]. In addition, the importance of host cDC in cross-presenting LAA was recently highlighted in a mouse model of post-transplant AML [125].

### 3.2. In Vivo Delivery of Antigen and Adjuvants to DCs

The delivery of leukemic antigen to DCs in vivo combines the advantages of avoiding the long and costly process of ex vivo moDC preparation and allowing the targeting of specific DC subsets [126]. Several considerations must be made when designing in vivo based vaccines. First, the right DC receptor must be identified; ideally this receptor is expressed by a DC subset which is present in sufficient numbers and fully functional during low disease burden or remission. In leukemia, the optimal DC receptor and subset for antibody targeting remains to be determined. Second, targeting to this receptor should result in antigen internalization and limited intra-endosomal destruction so that antigen may be cross-presented [127]. Third, the method of delivery must be intrinsically immunogenic or adjuvants must be co-delivered to avoid T cell tolerance and overcome immune suppression by the tumor [128,129]. Lastly, the method of delivery must not interfere with the DC’s ability to travel to lymph nodes to prime T cells [130]. Currently, in vivo vaccination approaches comprise DC-targeting antibodies, viral antigen-delivery systems, and organic molecular or nanoparticle-based approaches.

#### Targeting Antibodies

Owing to the quantitative and functional deficits observed in various DC subsets in many leukemias, antibody-mediated antigen delivery is unlikely to be effective during active disease. However, several groups have reported that cDC and moDC phenotype and function are largely restored during remission in AML [34,53], CLL [73], and ALL [65], suggesting that DCs may be targeted in vivo to eliminate residual leukemic cells. The administration of agents such as Flt3-L to expand circulating cDCs and enhance responses to targeting antibodies could be considered in patients whose tumours do not express the Flt3 receptor, as this technique runs the risk of expanding residual leukemic cells. The co-delivery of adjuvant is necessary for all DC targeting antibodies to avoid the induction of tolerance by DCs as demonstrated in a mouse model of AML [37], or biased CD4^+^ responses to a T follicular helper cell phenotype when using targeting antibodies alone [131].

**Figure 4:**
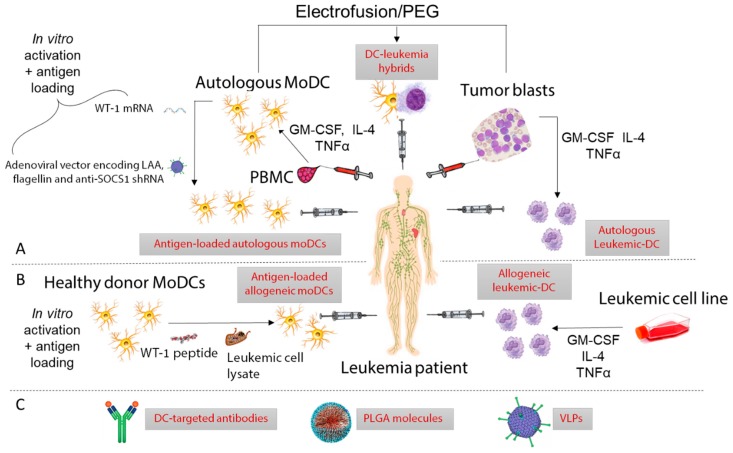
Summary of recent clinical trials and preclinical vaccination strategies utilizing dendritic cells to vaccinate leukemia patients. (**A**) Ex vivo autologous approaches: Autologous moDC derived in remission from patient PBMC have been used extensively in AML patients with the aim of preventing relapse. Promising results have been obtained by Wang and van Tendeloo et al. by loading DCs with WT-1 mRNA [105,113] or transfecting them with modified adenovirus [105]. Leukemic-DC have also been used by Westermann and Ossenkoppele et al. by isolating patient blasts and maturing to DC with cytokines [87,88]. Autologous hybrids fused using polyethylene glycol (PEG) or electroporation combining the antigen-presenting capacity of moDC with the neoantigen repertoire of leukemic blasts have been tested by Rosenblatt et. al, achieving exciting clinical results [100]. (**B**) Ex vivo allogeneic approaches: Allogeneic moDC derived from healthy donors and loaded with tumor cell lysate or WT-1 peptide have been used by Hus and Shah et al. [95,108]. Promising results have been achieved by van Loosdrecht et al. who matured an AML cell line with cytokines to allogeneic AML-DC before injection into AML patients [93]. (**C**) Several vaccination strategies exist for targeting DCs in vivo. Zhang et al. were able to induce an immune response in vitro using WT-1 loaded PLGA molecules which were phagocytosed by DCs [142], and Griffiths et al. induced immune responses in AML patients vaccinated with DEC-205 antibodies [137]. Virus-like particles (VLPs) are yet to be tested in the clinic.

Antibodies specific for DC receptors have already been used to deliver antigens and adjuvants to DCs in mouse models and in clinical trials for a range of diseases [132]. Targeted receptors include the C-type Lectin Receptors (CLRs) DEC-205 (CD205) [133], CLEC9A [134], DC-SIGN [135], as well as the chemokine receptor XCR1 [136]. Among them, DEC-205 has received most attention in the cancer immunotherapy setting. In a phase I clinical trial, anti-DEC-205 Abs conjugated to the tumor antigen NY-ESO-1 were found to induce antigen-specific T cell responses in a subset of patients with solid malignancies [133]. Another clinical study utilizing DEC-205 antibodies conjugated to NY-ESO-1 co-administered with the immune adjuvant PIC and decitabine chemotherapy detected NY-ESO-1-specific CD4^+^ and CD8^+^ T cells associated with the presence of CD141^+^ cDC1s in AML and myelodysplastic syndrome patients [137]. This result suggests that cDC1s are critically implicated in the generation of anti-leukemic T cells. Importantly, DEC-205 is expressed by a range of cell types other than cDC1s [138], suggesting that the specific targeting of cDC1s may facilitate priming of a greater number of T cells. Thus, numerous studies have advocated the use of the cDC1 specific CLR CLEC9A as a vaccine target in cancer owing to its selective expression and involvement in cross-presentation [37,131,134,139,140]. Tullett et al. have used humanized mice to demonstrate that a human chimeric anti-CLEC9A Ab specifically and efficiently delivers antigens to human CD141^+^ DCs in vivo [134]. In this study, CLEC9A-targeted antigens were efficiently presented by CD141^+^ DCs to CD4^+^ and CD8^+^ T cells. Therefore, CLEC9A-targeting Abs may confer therapeutic advantage over DEC-205-targeting Abs [141], although this remains to be demonstrated in clinical trials. A summary of several clinical and pre-clinical vaccination strategies in leukemia is shown in Figure 4 and Supplementary Table S1.

### 3.3. Considerations for the Design of Future Vaccines

Although the optimal timing for DC vaccination has not been established, most researchers agree that DC vaccination as initial therapy in leukemia is unlikely to be feasible due to immunologic alterations and the dysfunction of the DC compartment in leukemic patients at diagnosis [143]. Promisingly, there is evidence that the cDC and moDC compartments make a functional recovery after disease burden is reduced in various leukemia subtypes [34,39,61,73], and immunogenic cell death of leukemic blasts following anthracycline treatment may enhance tumour cell recognition and engulfment by DCs [144,145]. However, the quantity, and functional status of circulating DCs and T cells need to be considered, even during chemotherapy-induced remission. Indeed, AML patients in remission who received fludarabine during induction chemotherapy demonstrate an abnormal T cell landscape associated with a reduced capacity to respond to vaccination [34]. As a result and in spite of these factors, several DC-based vaccines have shown promising clinical outcomes when administered after initial therapies such as chemotherapy or allogeneic SCT in AML [93,100,104,105]. For ex vivo generated cellular vaccines, autologous PBMC have been harvested during complete or partial remission [100,104], whereas allogeneic PBMC from healthy donors or DCs from cell lines can be harvested at any time to be administered to patients in remission [93,105].

Newly improved DC-based therapy should be designed to eradicate minimal residual disease in remission patients. Given the very dismal prognosis of AML, most of the efforts have been and are still focused on this malignancy. AML is the focus of ~70% of the clinical trials harnessing DCs in leukemia. In other leukemias, the advent of highly effective targeted treatments such as imatinib in CML has markedly increased response rates, and the mortality rate for CML is now only 1–2% per year [146]. However, the financial burden and side-effects associated with lifelong adherence to tyrosine kinase inhibitors (TKIs) may provide a rationale for additional therapies including DC vaccination in the future. Targeted antibodies and chimeric-antigen-receptor (CAR) T cells have also increased response rates in lymphoid leukemias, but not to the level of TKIs in CML [110,147]. 5-year OS for ALL and CLL are approximately 39% [148] and 60% [149] respectively, allowing room for the development of novel therapies.

Combining DC vaccination with CAR T cell therapy is an interesting prospect suggested by multiple groups in both DC [150] and CAR T cell fields [151], as the efficacy of adoptive T cell therapy in mouse models of solid tumours has been correlated with the presence of intra-tumoral murine cDC1s which secrete high levels of CXCL9 and CXCL10 to attract T cells to the tumour site [152,153]. This effect may be dispensable in the context of active leukemia, as tumour cells are disseminated in the patient’s blood, but the activation of tumour-specific T cells in the BM may serve to eradicate long-lived leukemic stem cells after chemotherapy. Most importantly, WT-1 peptide-loaded DCs were shown to expand and activate CAR T cells in a K562 xenograft mouse model [154], providing rationale for the combination of CAR T cells and DC vaccination in the clinic. The forced expression of IL-7 and CCL19 by CAR T cells is another attractive strategy to enhance clinical responses as it leads to greater tumour infiltration by DCs and increased survival in mouse models of solid tumours expressing CD20 [155]. However, a major limiting factor in the dual clinical application of CAR T cells and DCs is the cost—the CD19-directed CAR T cell therapy tisagenlecleucel currently costs approximately US$475,000 per patient alone [156], and the only FDA approved vaccine containing DCs (Provenge) is approximately $93,000 per patient. It is important that future DC vaccines are able to be mass produced in a streamlined manner to reduce cost to patients and healthcare systems.

The combination of DC-based therapy with immune checkpoint inhibitors is another appealing strategy [157]. Depending on the immune checkpoint targeted, such combination could either allow better priming of T cell responses through the DC vaccine (e.g., if combined with anti-CTLA4 mAbs) or enhance/prevent exhaustion of the vaccine-induced responses (e.g., if combined with anti-PD-1 mAbs). In other words, DC vaccines would ensure the antigen-specificity of the response while checkpoint blockade would further amplify this response. The hypothesis of a synergy between checkpoint blockade and DC vaccination is supported by preliminary in vitro data [34,158]. A clinical trial has been set up to investigate the efficacy of vaccination DC-tumor hybrids combined with PD-1 blockade in AML (NCT01096602). Moreover, genetic engineering might be used to silence immune checkpoints within the DC vaccine [159,160] and a clinical trial is underway evaluating PD-L1/L2 silenced moDC in the post-transplant setting for a range of hematological malignancies (NCT02528682). However, while avoiding the immune-related adverse events caused by checkpoint inhibitor mAbs [161], this approach may prove less efficient as it will not prevent the inhibition of T cells by ligands expressed on leukemic cells [162,163,164,165,166].

## 4. Conclusions

Two decades after a DC vaccine was administered for the first time to a leukemic patient [86], DC vaccination in leukemia has finally taken off with the report of four very promising clinical trials [93,100,104,105]. There is now strong evidence that DC-based therapies can prevent or at least delay relapse in AML patients achieving remission. Recent technical progresses allowing specific targeting of the cDC1 subset as well as combinations with immune checkpoint blockades and CAR T cells are likely to tremendously enhance the efficacy of DC-based approaches. Therefore, we remain hopeful that DC-based therapy might improve the fate of leukemia patients in a near future.

## Supplementary Materials

The following are available online at https://www.mdpi.com/2072-6694/11/6/875/s1, Table S1: Selected clinical trials and preclinical data for ex vivo DC vaccination in Leukemia.
