# Supplementary material for: Can Dendritic Cell Vaccination Prevent Leukemia Relapse?
Source: Cancers (Basel). 2019 Jun 22;11(6):875. doi: 10.3390/cancers11060875 (PMC6627518; doi:10.3390/cancers11060875)
Supplement: Supplementary file 1 [file cancers-11-00875-s001.pdf]

Supplementary Materials

Can Dendritic Cell Vaccination Prevent Leukemia Relapse?

Liam J. O'Brien, Camille Guillerey and Kristen J. Radford

Table S1. Selected clinical trials and preclinical data for ex vivo DC vaccination in Leukemia. 5-year OS for AML is approximately 24%, 5-year OS for CML is approximately 68%, 5 year OS for ALL is approximately 71%, 5 year OS for CLL is approximately 83% (Society, 2018).

Disease	Reference	Phase	Type of DCs Used	Activation/Ag Loading Method	Injection Method	Outcome/s
AML	NCT01734304 [103]	Pilot	Autologous MoDC	$CD14^+$ cells cultured with GM-CSF, IL-4 (48 h) + R848, TNFα, IL1β, IFNγ, PGE2 (24 h). Electroporated with mRNA encoding 3 different TAAs (WT1, PRAME, hCMVpp65) in 3 aliquots. MoDCs then combined for a mixed population.	15×10^6 DCs intra-dermally weekly for 4 weeks then monthly up to 6 months in post-remission patients.	Autologous T cell responses in humanized NSG mice (enhanced compared to 7day moDC maturation methods), ongoing clinical trial in human patients
AML	[141]	I	AML-DC	MNC differentiated to DC with GM-CSF, TNFα, IL-3, Flt3-L and IL-4 or CI A23187 and IL-4.	4 intradermal injections of AML-DCs weekly for 4 weeks in relapsed AML patients in 2nd complete remission. KLH and GM-CSF administered with vaccine.	n = 15<br>no DC vaccinations completed as patients did not achieve complete remission.
AML	NCT03059485 [100]	II	Autologous moDC fused with AML cells	Adherent cells cultured with GM-CSF and IL-4 (5–7 days) + TNFα (2–3 days). DC and AML cells then cocultured with PEG to generate hybrids.	3 doses of 5×10^6 fusion cells administered monthly to remission patients	n = 17<br>71% OS at median follow up of 57 months. Extremely positive outcome.
AML	NCT00965224 [104]	II	Autologous MoDC	$CD14^+$ cells cultured with GM-CSF, IL4 (6 days), + PGE2, TNFα, KLH (2 days). Electroporated with mRNA encoding WT-1.	4 biweekly intradermal injections of 5, 10, or 20×10^6 DCs in the upper arm of post-remission patients.	n = 30<br>PR 13%, CR 30%. 5-year OS 53.8% in responders, 25.8% in non-responders (40% overall). Long term response correlated with increased circulating frequencies of WT-1 specific CTLs. Positive outcome.
AML	NCT03697707 [93]	I	Allogeneic DC from AML cell line MUTZ-3	MUTZ-3 cells cultured with GM-CSF, TNF-α IL4, mitoxantrone (5–7 days), + PGE2, IL1β (1 day)	4 biweekly intradermal injections of 10, 25 or 50 million cells in advanced-stage elderly patients	n = 12<br>50% CR at study end (2 years), 42% had progressive disease. Long term survival correlated with maintained T cell levels. Positive outcome.

AML & ALL	NCT01956630 [105]	II	Genetically modified moDC in combination with cytokine induced killer cells	Adenoviral vector encoding SOCS1 shRNA, surviving, MUC1, and flagellin sequence fragments used to infect DCs generated from allogeneic donor PBMC with GM-CSF and IL-4 (2 days)	Relapsed patients after aHSCT received 4 s.c. injections of 2–5 × 10^7 gmDCs in the groin, axilla and neck on days 7, 9, 11 and 13, followed by 2 infusions of cytokine-induced killer cells (>10^9)	*n* = 23 for gmDC, *n* = 25 for DLI.<br>gmDC group: 57% CR, 3-year OS 48.9%, significant reduction of severe (grade 3–4) aGvHD compared to DLI after aHSCT (0/25 vs. 9/23).<br>DLI: 48% CR, 27.5% OS at 3 years.<br>Positive outcome.
AML, ALL, HL	NCT00923910 [108]	Pilot	Allogeneic moDCs in combination with DLI	$CD14^+$ cells cultured with GM-CSF and IL-4 (3 days) + LPS and IFNγ (1 day)	Relapsed patients after aHSCT received 1 × 10^6 $CD3^+$/kg once every 4 weeks × 3 and the same dose of DC biweekly × 6.	*n* = 4<br>100% PD by end of study.<br>Poor outcome.
CML	[87]	I	CML-DC	GM-CSF, IL-4, TNF*α* (3 weeks), KLH (2 h)	2–3 × 10^6 CML-DCs weekly for 4 weeks intradermally on right anterior thigh	*n* = 3<br>Elevated IFNγ release by $CD4^+$ T cells after vaccination. DTH reactions against CML cells in 2/3 patients 20 months after vaccination. Study stopped early due to availability of TKI
CML	[89]	I	CML-DC	Not available	Four injections of 3 × 10^6 DC and 15 × 10^6 DC.	*n* = 6<br>No clinical responses. T cells drawn later in course of therapy were more sensitive to stimulation by CML-DC *in vitro*<br>Poor outcome.
CML	[88]	I/II	MoDC	$CD14^+$ cells cultured with GM-CSF, IL4 (5 days), + TNF-*α* (3-4 days) + KLH (3-4hrs)	S.c. injection (inguinal) days 1, 2, 8, 21 using increasing doses of DCs (1 × 10^6 d1, 50–100 × 10^6 d21)	*n* = 10<br>Cytogenetic response in 4/10 patients, expansion of leukemia-specific T cells in 3/10 patients.<br>20%PD, 60%SD, 10%excluded<br>Positive outcome.
B-ALL	[142]	Pre-clinical data	DC-like cells from ALL blasts	$CD19^+$ cells cultured with IL-4 (5 days) + irradiated CD40L-transfected L cells (2 days)	**N/A**	Expressed costimulatory molecules and induced proliferative responses in naïve $CD4^+$ T cells
B-ALL	[143]	Pre-clinical data	DC-like cells from ALL blasts	$CD19^+$ cells cultured with IL-1β, IL-3, IL-7, SCF, TNF-*α* and CD40L	**N/A**	4/5 cell lines and 3/3 patients exhibited DC-like differentiation of blasts. B-DCs showed CD80, CD86, CCR7 expression, and were able to stimulate T cell proliferation in MLR.
CLL	EudraCT nr 2010-024224-18 [106]	I	MoDC + Lenalidomide	Cultured with GM-CSF, IL-4 (3 days) + TNF-*α*, irradiated leukemia cells (2 days)	Intradermal injection of 20 × 10^6 DCs at week 0, 2, 4, 6, 14.	*n* = 10<br>Dose-limiting toxicity in 30% of patients.<br>Vaccine-induced immune responses in 90% patients. 30% PR, 60% SD, 10% PD<br>Good outcome.

CLL	[96]	I	MoDC	Cultured with GM-CSF, IL-4 (3 days) + TNF-α, leukemia cell lysates (2 days)	Early-stage patients intradermally injected with DCs at 2–4 weeks intervals, repeated 5–8 times	n = 12<br>42% PR, 33% SD, 25% PD<br>Good outcome.
CLL	[111]	I	MoDC cultured with apoptotic bodies	Cultured with GM-CSF + IL-4 (3 days) + apoptotic B cells on day 4 + TNF-α (day 5–7)	>1 × 10^7 DCs intradermally injected at weeks 0, 2, 4, 6, 14 in upper arm.	n = 15<br>No objective clinical responses, but 60% patients displayed immune responses in IFNγ ELISPOT and CD107 degranulation assay.<br>Poor outcome.

Abbreviations: DLI: Donor Lymphocyte Infusion, PR: Partial response, CR: Complete response, OS: Overall Survival, PD: Progressive Disease, B-DC: Malignant B-cell derived DCs, PEG: Polyethylene glycol, gmDC: Genetically-modified DC, aGvHD: acute Graft vs. Host Disease, SOC: Standard-of-care.

© 2019 by the authors. Licensee MDPI, Basel, Switzerland. This article is an open access article distributed under the terms and conditions of the Creative Commons Attribution (CC BY) license (http://creativecommons.org/licenses/by/4.0/).
